# Prediction of hearing outcomes in chronic otitis media patients underwent tympanoplasty using ossiculoplasty outcome parameter staging or middle ear risk indices

**DOI:** 10.1371/journal.pone.0252812

**Published:** 2021-07-29

**Authors:** Da Jung Jung, Hyun Ju Lee, Ji Song Hong, Dong Gyu Kim, Jae Yeon Mun, Jong-Won Bae, Myung Hoon Yoo, Kyu-Yup Lee

**Affiliations:** Department of Otorhinolaryngology-Head and Neck Surgery, School of Medicine, Kyungpook National University, Daegu, Republic of Korea; Universidade Federal de Sao Paulo/Escola Paulista de Medicina (Unifesp/epm), BRAZIL

## Abstract

**Purpose:**

Ossiculoplasty outcome parameter staging (OOPS) and middle ear risk index (MERI) are the most commonly used indices for predicting prognosis of patients with chronic otitis media (COM). This study aimed to verify the efficiency of OOPS and MERI scores in predicting outcomes of patients with COM who underwent tympanoplasty.

**Methods:**

We retrospectively reviewed the data of patients who underwent tympanoplasty (n = 526). OOPS, and MERI scores were collected. Hearing data were measured 1 day preoperatively, and 3 and 12 months postoperatively. Operation success was defined according to the Korean Society of Otology guidelines.

**Results:**

For calculation of success, the ROC values of MERI were 0.551 at 12 months. ROC values of OOPS were 0.637 at 12 months. There were no significant differences in hearing variables among the three groups according to MERI. There were significantly favorable outcomes in hearing variables in the low-risk group in OOPS. The mean OOPS score was greater in patients with success than those with non-success. Otorrhea, ossicle status, and status of mucosa as variables in both indices were associated with success. The type of mastoidectomy as a variable in OOPS alone was associated with success. Absence of hypertension, presence of ossiculoplasty, and use of incus as ossiculoplasty material were associated with poor success rate.

**Conclusion:**

Compared with MERI, the OOPS index was more closely associated with the hearing outcomes, which may be due to the extent of inflammation in the OOPS index.

## Introduction

Chronic otitis media (COM) is a well known ear disease globally. The prevalence of COM in the United Kingdom or South Korea is 4.1% and 3.8%, respectively [1, 2]. Although the variations in degrees exist, most patients with COM are associated with the development of hearing impairment. There are many factors that can affect the outcomes including function of the Eustachian tube, middle ear conditions, types of surgical interventions, status of residual ossicular remnants, and type of operation or prosthesis [3]. Staging or index systems using significant variables may be useful to predict prognosis in patients with COM.

Ossiculoplasty outcome parameter staging (OOPS) and middle ear risk index (MERI) are the most commonly used indices for predicting prognosis of patients with COM or ear operation. In 1994, Kartush et al. initially suggested a scoring system termed MERI, and Becvarovski and Kartush revised the final system, including the smoking status [4, 5]. However, Dornhoffer et al. found a non-linear association between MERI and hearing outcomes in patients with more extensive pathologic conditions and published OOPS as a new staging system [6]. The indices share some variables such as otorrhea, ossicular status, presence of surgical intervention, and mucosal status of the middle ear; however, the presence of cholesteatoma, perforation of the tympanic membrane, smoking, and type of surgical intervention are not common between them. In addition, possible factors (age, underlying comorbidities, and type or materials of ossiculoplasty), which may be associated with prognosis, are not included in the two indices. Therefore, further data are needed to identify the clinical significance of each indicator, additional prognostic factors except indicators within the two indices, and superiority in predicting prognosis between them. This study aimed to verify the efficiency of OOPS and MERI scores in predicting outcomes of patients with COM who underwent tympanoplasty and the association between each factor within the indices and hearing results. Further, we aimed to evaluate other possible factors that affect hearing outcomes.

## Materials and methods

### Patient selection

We retrospectively reviewed the clinical data of 614 patients who underwent tympanoplasty at Kyungpook National University Hospital between January 2017 and December 2018. In our study, the first date of the participant’s medical information was January 1, 2017, and the authors accessed medical records between September 1, 2019, and August 31, 2020. All the operations in our study were performed by three surgeons with more than 7 years of experience in performing tympanoplasty. We excluded patients aged < 18 years, those with incomplete data, those with < 1 year of follow-up duration, those with preoperative profound hearing loss, or those with cholesteatoma beyond the middle ear and the mastoid cavity, and 526 participants were included in the analysis.

The following baseline demographic and clinical characteristics were collected: age at operation, sex, follow-up duration, presence of diabetes mellitus or hypertension, materials for ossiculoplasty, status of eustachian tube, OOPS, and MERI scores, pre- and post-operative speech discrimination test (SDT), postoperative air-bone gap (ABG), air gain (AG), pre- and post-operative air conduction (AC), ABG closure, and change of high tone bone conduction (△Ht-BC).

In our center, tympanoplasty alone was performed in patients with perforation of tympanic membrane, no abnormalities in ossicle mobility, and no evidence of soft tissue density in the middle ear and the mastoid cavity using preoperative computed tomography. Tympanoplasty with ossiculoplasty was performed in patients with fixation or discontinuity in ossicle mobility and no evidence of soft tissue density in the mastoid using preoperative computed tomography. Tympanoplasty with mastoidectomy was performed in patients with evidence of soft tissue density in the middle ear and the mastoid using preoperative computed tomography and/or with purulent discharge and otorrhea for ≥ 3 months. The eustachian tube opening was directly inspected during the operation to check if it had a mucosal web or was obstructed; this was later recorded. The study was approved by the Kyungpook National University Hospital Institutional Review Board (KNUH 2020-06-058). The board waived the need for informed consent.

### OOPS and MERI scoring

In our center, we did not routinely calculate the OOPS and MERI scores; however, most data for the two scores were independently evaluated and recorded in medical charts. We retrospectively analyzed these data and calculated the two scores. All patients had data for the two scores and outcomes. Some did not have the data for these, but the patients were excluded in our study. The OOPS and MERI scores were calculated using a previously described method [5, 6]. Briefly, OOPS includes otorrhea, perforation of the tympanic membrane, the presence of cholesteatoma, ossicular status, status of the middle ear, previous surgery, and smoking. MERI includes otorrhea, ossicular status, status of mucosa, previous surgery, and presence/type of mastoidectomy. S1 Table shows the scoring system of the two indices. For MERI, scores of 1–3, 4–6, and 7–12 were defined as mild, moderate, and severe groups, respectively [7]. For OOPS, scores of 1–3, 4–6, and 7–9 were defined as low, intermediate, and high-risk groups, respectively [7].

### Audiometric methods

Hearing data were measured 1 day preoperatively, and 3 and 12 months postoperatively. The data included pure tone air and bone conduction thresholds at 0.5, 1, 2, 3, 4, 6, and 8 kHz. The data were calculated according to the 1995 American Academy of Otolaryngology–Head and Neck Surgery, Committee on Hearing and Equilibrium consensus guidelines [8, 9]. Pure tone average thresholds were divided into four frequencies; 0.5, 1, 2, and 3 kHz in the speech range. The postoperative ABG was calculated as the difference between postoperative AC and preoperative bone conduction (BC). AG was calculated as the difference between preoperative and postoperative AC values. A △Ht-BC was defined as the difference between preoperative and postoperative high pure-tone BC at 1, 2, and 4 kHz, as suggested by the guidelines. ABG closure was calculated as the difference between preoperative and postoperative ABG values. Operation success was defined according to the Korean Society of Otology guidelines as more than one of the following conditions: postoperative ABG ≤ 20 dB, AG ≥ 15 dB, or postoperative AC < 30 dB. The placement of the intact graft at the correct place was also evaluated and defined as a condition without tympanic membrane perforation, extrusion of prosthesis, and postoperative inflammation. The graft was evaluated by endoscopy at 2 weeks and, 1, 3, 6, and 12 months after the operation.

### Statistical analyses

Data were analyzed using SPSS version 21 (IBM Corp., Armonk, NY, USA). Categorical data are expressed using frequencies and percentages, and continuous data are expressed as means ± standard deviations. Categorical data were compared using the chi-square test and continuous data using Student’s t-test. Correlations were analyzed to assess the strength of the associations between continuous variables. The receiver operating characteristic (ROC) curve was used to calculate the probability of predicting success, cutoff values, sensitivity, and specificity. MedCalc version 11.6.1.0. (MedCalc, Mariakerke, Belgium) was used for ROC analysis. A *P*-value < 0.05 was considered statistically significant.

## Results

### Baseline characteristics

**Fig 1:**
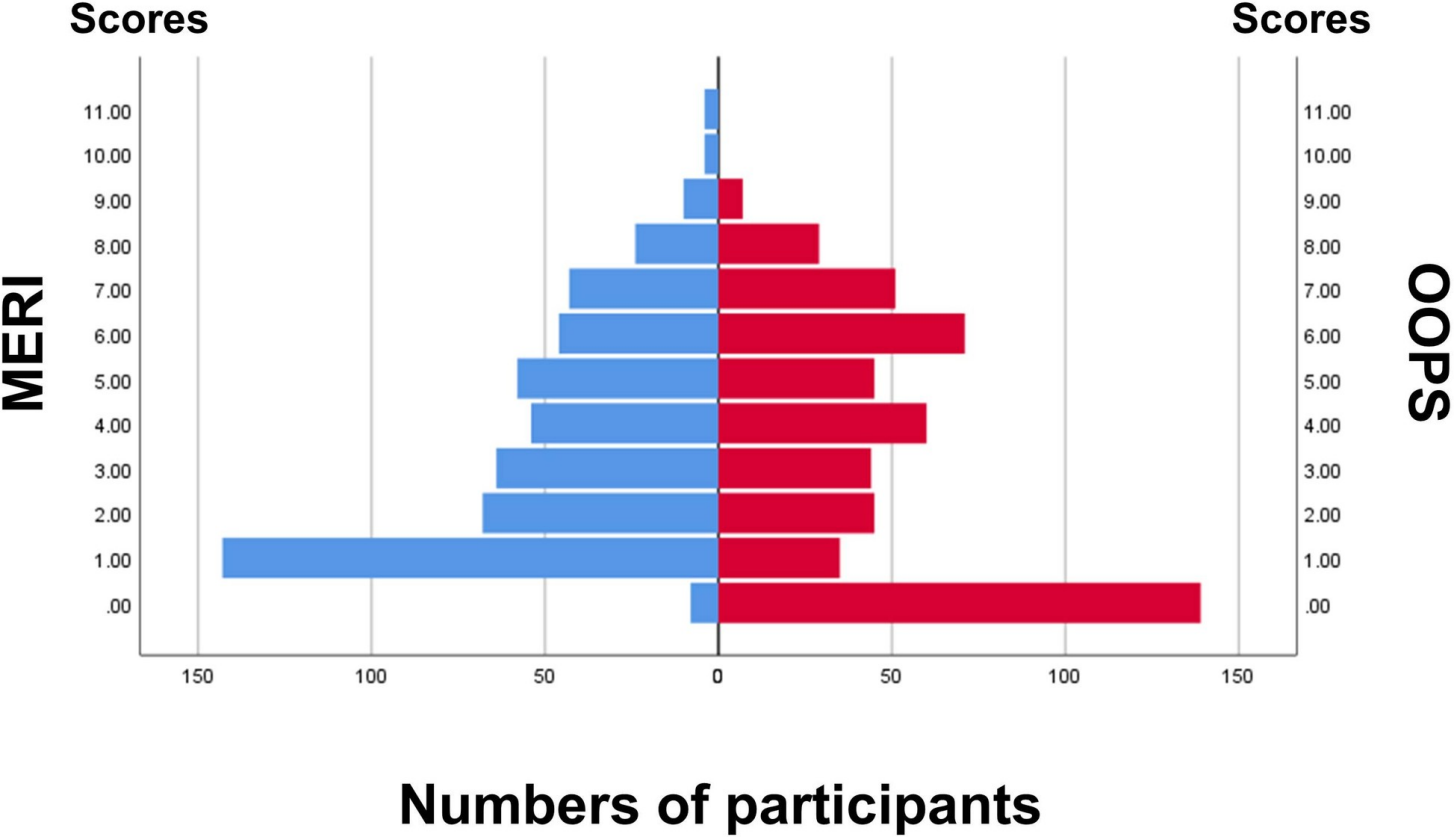
Histogram of MERI and OOPS scores. MERI, middle ear risk index; OOPS, ossiculoplasty outcome parameter staging.

Our cohort predominantly comprised women, and the mean age was 52.3 ± 15.4 years (Table 1). The number of patients who underwent ossiculoplasty and mastoidectomy was 300 (57%) and 294 (55.9%), respectively. Fig 1 shows the distribution of MERI and OOPS scores. The mean scores in MERI and OOPS were 3.7 ± 2.5 and 3.4 ± 2.8, respectively. The number of patients in the mild, moderate, and severe MERI groups was 283, 158, and 85, respectively, whereas those in the low, intermediate, and high OOPS groups was 263, 176, and 87, respectively. The proportions of the three groups in the two indices are shown in S1 Fig. The number of participants with re-perforation of the tympanic membrane, postoperative inflammation, or extrusion of the prosthesis were 14 (2.7%), 4 (1.6%), and 1 (0.2%), respectively.

**Table 1:** Baseline characteristics.

Variables	n = 526
Age (years)	52.3 ± 15.4
Sex (men)	212 (40.3%)
Side (right)	244 (46.4%)
Follow-up duration (months)	17.0 ± 6.1
Diabetes mellitus	44 (8.4%)
Hypertension	105 (20.0%)
Smoker	
Non-smoker	508 (96.6%)
Smoker	18 (3.4%)
Ossiculoplasty	
None	221 (42.0%)
Columellization	275 (52.3%)
Interposition	30 (5.7%)
Preoperative SDT	92.7 ± 20.1
Preoperative AC	49.4 ± 22.0
Preoperative BC	27.2 ± 16.8
Preoperative ABG	22.3 ± 11.2
Presence of cholesteatoma	133 (25.3%)
Fibrotic mucosa	252 (47.9%)
Ossicle status	
Malleus (erosion)	180 (34.2%)
Incus (erosion)	222 (42.2%)
Stapes (erosion)	99 (18.8%)
Mastoidectomy	
None	232 (44.1%)
CWU	75 (14.3%)
CWD	219 (41.6%)
Type	
Type 3	238 (45.2%)
Type 4	67 (12.7%)
Obstructed eustachian tube	109 (20.7%)
Revision operation	93 (17.7%)

The data are expressed as numbers (percentage) for categorical data and mean ± standard deviation for continuous data.

SDT, speech discrimination test; AC, air conduction; BC, bone conduction; ABG, air-bone gap; CWU, canal wall up; CWD, canal wall down.

### Association between hearing outcomes and two indices

**Fig 2:**
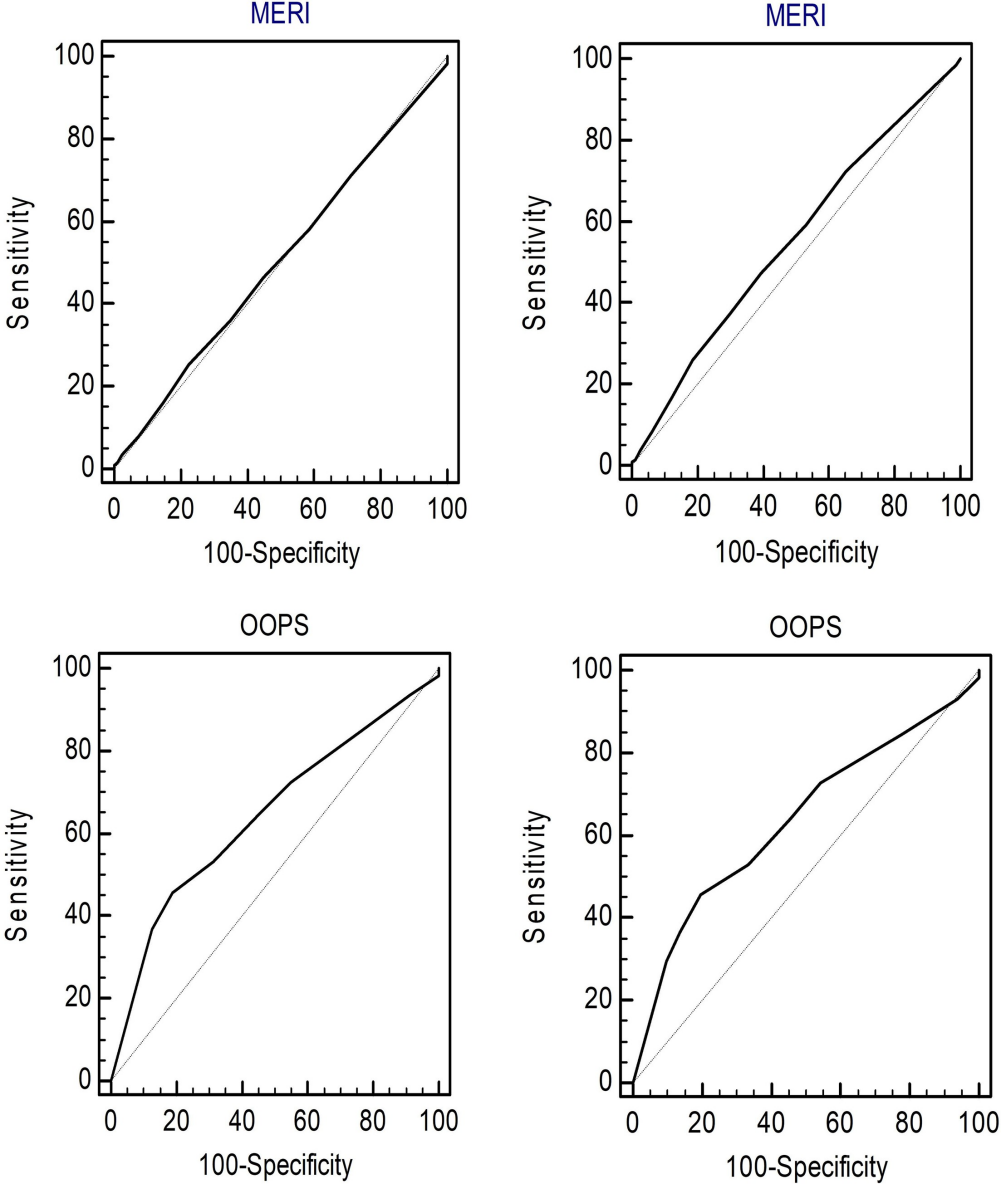
ROC curves for two indices for predicting success at 3 or 12 months after ossiculoplasty. (A) ROC curve for MERI for predicting success at 3 months, (B) ROC curve for MERI for predicting success at 12 months, (C) ROC curve for OOPS for predicting success at 3 months, (D) ROC curve for OOPS for predicting success at 12 months. MERI, middle ear risk index; OOPS, ossiculoplasty outcome parameter staging; ROC, receiver operating characteristic.

The number of successes at 3 and 12 months after surgery was 446 (84.8%) and 445 (84.6%), respectively. Fig 2 shows ROC curves for predicting the success of the two indices. For calculation of success, the ROC values of MERI were 0.504 (95% confidence interval [CI], 0.461–0.548; *P* = 0.895) at 3 months and 0.551 (95% CI, 0.508–0.594; *P* = 0.130) at 12 months. ROC values of OOPS were 0.645 (95% CI, 0.602–0.686; *P* < 0.001) at 3 months and 0.637 (95% CI, 0.594–0.678; *P* < 0.001) at 12 months.

The number of successes in the mild, moderate, and severe MERI groups was 239 (84.5%), 134 (84.8%), and 73 (85.9%), respectively, at 3 months and 234 (82.7%), 136 (86.1%), and 75 (88.2%), respectively, at 12 months (*P* = 0.949 for 3 months and *P* = 0.382 for 12 months). The number of successes in the mild, moderate, and severe OOPS groups was 238 (90.5%), 140 (79.5%), and 87 (78.2%), respectively, at 3 months and 236 (89.7%), 140 (79.5%), and 69 (79.3%), respectively, at 12 months (*P* = 0.001 for 3 months and *P* = 0.005 for 12 months). The number of cases with intact grafts in the mild, moderate, and severe MERI groups was 277 (97.9%), 147 (93.0%), and 83 (97.6%), respectively. Further, those in the low, intermediate, and high OOPS groups was 249 (94.7%), 173 (98.3%), and 85 (97.7%), respectively. There were no significant differences in six hearing variables (postoperative ABG, ABG closure, △Ht-BC, AG, postoperative AC, and postoperative SDT) among the three groups according to MERI (S2 Table). There were significantly favorable outcomes in four hearing variables (postoperative ABG, AG, postoperative AC, and postoperative SDT) in the low-risk group in OOPS.

Correlation analysis showed that OOPS alone was positively associated with postoperative ABG, △Ht-BC, AG, and postoperative AC and inversely associated with postoperative SDT (Table 2). The mean MERI score of patients with success or non-success was 3.7 ± 2.5 and 3.6 ± 2.4 at 3 months and 3.7 ± 2.5 and 3.3 ± 2.4 at 12 months, respectively (*P* = 0.808 at 3 months and *P* = 0.139 at 12 months). The mean OOPS score of patients with success or non-success was 3.2 ± 2.8 and 4.6 ± 2.4 at 3 months and 3.2 ± 2.8 and 4.5 ± 2.4 at 12 months, respectively (*P* < 0.001 at 3 months and *P* < 0.001 at 12 months).

**Table 2:** Correlation coefficients between two indices and hearing outcomes.

	Postop ABG	*P*	ABG closure	*P*	△Ht-BC	*P*	Air gain	*P*	Postop AC	*P*	Postop SDT	*P*
3 Months												
OOPS	0.344	<0.001	–0.024	0.578	0.092	0.035	0.168	<0.001	0.404	<0.001	–0.205	<0.001
MERI	0.007	0.871	0.010	0.811	0.053	0.228	0.012	0.780	0.056	0.201	0.009	0.832
12 Months												
OOPS	0.303	<0.001	–0.015	0.730	0.066	0.130	0.138	0.001	0.394	<0.001	–0.206	<0.001
MERI	0.013	0.768	0.005	0.902	0.036	0.407	0.040	0.357	0.053	0.224	0.003	0.757

The data are expressed as correlation coefficients.

Postop; postoperative, ABG; air-bone gap, △Ht-ABG; change in high-tone bone conduction, AC; air conduction, SDT; speech discrimination test, OOPS; ossiculoplasty outcome parameter staging, MERI; middle ear risk index.

### Association between hearing outcomes and each variable

Smoking status as a variable in MERI alone was associated with success (Table 3). Otorrhea, ossicle status, and status of mucosa as variables in both indices were associated with success. The type of mastoidectomy as a variable in OOPS alone was associated with success. We evaluated the association between success and age, the eustachian tube status, diabetes mellitus, hypertension, and presence/type of ossiculoplasty as variables without two indices (S3 Table). Absence of hypertension, presence of ossiculoplasty, and use of incus as ossiculoplasty material were associated with poor success rate.

**Table 3:** Comparisons of success rate according variables in two indices.

	Total numbers	Success (3M)	P-value	Success (12M)	P-value
**Variables in MERI alone**					
Perforation			0.154		0.057
Absence	131	106 (80.9%)		104 (79.4%)	
Presence	395	340 (86.1%)		341 (86.3%)	
Cholesteatoma			0.107		0.673
Absence	393	339 (86.3%)		334 (85.0%)	
Presence	133	107 (80.5%)		111 (83.5%)	
Smoking			0.004		0.032
Non-smoker	508	435 (85.6%)		433 (85.2%)	
Smoker	18	11 (61.1%)		12 (66.7%)	
**Variables in OOPS alone**					
Mastoidectomy			< 0.001		< 0.001
No	232	216 (93.1%)		214 (92.2%)	
CWU	75	63 (84.0%)		63 (84.0%)	
CWD	219	165 (75.3%)		167 (76.3%)	
**Variables in both MERI and OOPS**					
Otorrhea			0.231		0.026
Absence	390	335 (85.9%)		338 (86.7%)	
Presence	136	111 (81.6%)		107 (78.7%)	
Ossicle			<0.001		<0.001
Normal	238	222 (93.3%)		218 (91.6%)	
Presence of malleus	112	76 (67.9%)		80 (71.4%)	
Absence of malleus	176	148 (84.1%)		147 (83.5%)	
Mucosa			0.062		0.007
Normal	274	240 (87.6%)		243 (88.7%)	
Fibrotic	252	206 (81.7%)		202 (80.2%)	
Revision			0.062		0.595
No	433	373 (86.1%)		368 (85.0%)	
Yes	93	73 (78.5%)		77 (82.8%)	

Data are expressed as numbers (percentages). Statistical analyses are performed using the chi-square test.

3M, 3 months after operation; 12M, 12 months after operation; MERI, middle ear risk index; OOPS, ossiculoplasty outcome parameter staging; CWU, canal wall up; CWD, canal wall down.

## Discussion

Our study showed that OOPS was a better predictor of success after tympanoplasty than MERI. OOPS scores also correlated with other hearing outcomes such as postoperative ABG, △Ht-BC, AG, postoperative AC, and postoperative SDT; however, MERI scores did not correlate with these outcomes. There was a significant difference in OOPS alone between patients with and without success. Among variables within two indices, smoking, otorrhea, ossicle status, status of mucosa, and presence/type of mastoidectomy were associated with success. Beyond two indices, presence of hypertension, absence of ossiculoplasty, and use of artificial material in ossiculoplasty were also associated with success.

Previous studies have shown the usefulness of two indices to predict prognosis in patients who underwent tympanoplasty and/or ossiculoplasty; however, most studies considered only surgical aspects as outcomes or included small sample sizes. A study comparing the two indices may be insufficient to conclude due to the small sample size. Therefore, additional studies are needed to evaluate the superiority of prediction for prognosis between MERI and OOPS or identify the other possible factors that affect hearing outcomes.

Our study included a relatively large sample size with long-term follow-up and evaluated comprehensive hearing data. In our study, almost all patients (96.4%) had an intact graft after surgery, which was a parameter for success in the surgical aspect. Therefore, we evaluated various hearing outcomes. The Korean Society of Otology recommended the requirement of the three indicators; postoperative ABG, AG, and postoperative AC to define success. However, the American Academy of Otolaryngology-Head and Neck Surgery consensus recommended the evaluation of postoperative ABG, ABG closure, and △Ht-BC [9]. Although we evaluated all these variables, success was defined according to the Korean guideline.

The proportions of the three groups in the two indices were different, as shown in S1 Fig. Our study showed that OOPS is a better index for predicting prognosis than MERI. These may reveal that some factors in indices (mainly OOPS) play an important role in predicting prognosis. Therefore, we evaluated the association between each variable and hearing outcome and identified that, among variables, smoking, otorrhea, ossicle status, mucosa status, and presence/type of mastoidectomy were associated with hearing outcomes. The presence of perforation or cholesteatoma and revision operation were not associated with hearing outcomes. These results reveal that the extent of inflammation, in contrast to the presence of perforation or cholesteatoma as simple lesions, is associated with hearing outcomes.

The type of graft and surgical techniques influenced the outcome of tympanoplasty, but the OOPS and MERI score systems did not include all the variables in the surgical approaches. Our study compared all surgical approaches and showed that tympanoplasty with ossiculoplasty (type 3 or 4) or mastoidectomy was associated with a poor success rate. The outcomes of columellization and interposition in ossiculoplasty were similar. The presence of mastoidectomy, in addition to tympanoplasty, was considered while calculating OOPS scores; however. The canal wall down type of mastoidectomy was associated with poorer outcomes than canal wall up mastoidectomy.

The presence of a cholesteatoma influences the prognosis after tympanoplasty. Detailed information for cholesteatoma was not included in the OOPS and MERI scoring systems. MERI calculation considered the presence of a cholesteatoma without extension in the scoring system; however, our data showed that a cholesteatoma was not associated with a low success rate; the extension of cholesteatoma had more of an impact on the surgical outcome. Previous attempts to categorize cholesteatomas rely on extension, which depends on the selection of surgical approach and outcome after tympanoplasty. In the two scoring systems, ossicular status indirectly reflects the extension of cholesteatoma. In our study, cholesteatoma extension was restricted at the mastoid and the middle ear cavity. Patients with cholesteatoma beyond the middle ear cavity and the mastoid were excluded due to the high risk of postoperative complications and poor preoperative/postoperative hearing. Additionally, our study included data on the surgical site and the hearing result up to 12 months after surgery. The graft or recurrence of cholesteatoma was evaluated using endoscopy up to 12 months after the operation. No patient suffered from recurrent or remnant cholesteatoma at 12 months after the operation, however, this is a shorter duration than that required for the identification of recurrent or remnant cholesteatoma.

Surgical time and the expertise of the operating surgeon also influence the prognosis of a patient after tympanoplasty. A previous study showed that surgery performed by experienced staff members is associated with better results [10]. Our data did not include the effect of the expertise of the surgeon on the outcome of tympanoplasty. All the surgeries in our study were performed by three surgeons with more than 7 years of experience in performing tympanoplasty. Most of the surgeries were performed by one surgeon with more than 15 years of experience in performing tympanoplasty. Intact graft placement was defined as a condition in which no postoperative tympanic membrane perforation, extrusion of the prosthesis, and inflammation occurred. Among 526 patients, 19 (3.6%) of participants had a non-intact graft. Among these, postoperative inflammation in four cases was resolved after antibiotics were prescribed for 7 days. Further studies with a longer follow-up duration may be more useful to identify postoperative complications. However, considering a low postoperative complication rate and no case of remnant or recurrent cholesteatoma, the expertise of the operating surgeon may not have influenced the outcome of surgery in our cohort.

In our study, the presence of hypertension, absence of ossiculoplasty, and ossiculoplasty materials were also associated with success. We suggest that patients with hypertension on medications such as antihypertensives, dyslipidemic or anti-platelet agents, exhibit pleiotropy through anti-inflammatory effects. These may be associated with favorable results in such patients. Our study also evaluated the impact of diabetes mellitus and hypertension on the outcome of tympanoplasty; however, comorbidities beyond these two may influence the success rate of surgery. Most studies either excluded patients with other comorbidities, included a small proportion of these patients, or did not analyze the effect of comorbidities. These comorbidities can be associated with non-otologic complications, such as pulmonary and, cardiovascular diseases. However, there are little data regarding the association between comorbidities and outcomes after tympanoplasty. Further studies regarding the association between various comorbidities, including diabetes mellitus or hypertension, and their impact on hearing outcomes of tympanoplasty are needed to elucidate the role of comorbidities. In addition, there might be a difference in outcome between hypertensive patients who were on medication and hypertensive patients who were not. Furthermore, the classification of antihypertensive medications, such as inhibitors of the renin-angiotensin-aldosterone system or calcium channel blockers, or the achievement of the target blood pressure can influence the outcome. For diabetic patients, differences according to their characteristics, such as the achievement of target blood glucose levels and method of glucose control (diet, exercise, oral hypoglycemic agents, and insulin), may be greater. Although these issues are beyond the scope of our study, they could be another interesting issue regarding their impact on the outcome according to the presence of medication or classification in hypertensive or diabetic patients. Unfavorable results for incus compared with hydroxyapatite or titanium may be because of the small number of patients who underwent ossiculoplasty using incus (n = 28).

As Becvarovski and Kartush introduced a revised version of the MERI scoring system that included smoking, some studies have validated its importance [5, 9, 11–13]. Pinar et al. enrolled 231 patients who underwent tympanoplasty. They evaluated the importance of MERI for predicting a successful operation and showed that the size of perforation, a healthy opposite ear, and a dry period were factors associated with the outcome of surgery that were beyond the variables of MERI [9]. Kumar et al. evaluated the original version of the MERI and showed a positive association between the MERI group and successful surgery in 50 patients [11]; however, their study included only a small number of participants. Shishegar et al. evaluated 200 patients who underwent tympanoplasty and showed that MERI is a useful tool for predicting the success of tympanoplasty [13]. Moerover, they showed the importance of the duration of dryness of the ear, status of the opposite ear, and types of surgical technique (intact canal wall, canal wall down, or non-mastoidectomy) as additional risk factors beyond the variables of the MERI. However, these studies defined the success of surgery using the surgical aspect alone, such as intact graft status and well-aerated middle ear, without considering variables for hearing.

Cox et al. validated OOPS in 68 children and 126 adults who underwent ossiculoplasty and evaluated the changes in ABG (defined success as the ABG closure ≤ 20 dB) [14]. They showed a positive association between the OOPS score and hearing outcomes of an operation, but they did not evaluate the impact of each among and beyond the variables of OOPS. Our study compared the two scoring systems using large sample size and evaluation of various hearing outcomes, such as postoperative ABG, ABG closure, AG, postoperative AC, and postoperative SDT beyond the surgical aspect. Additionally, the absence of hypertension, the need for ossiculoplasty along with tympanoplasty, and the materials used for ossiculoplasty, which were beyond the variables of the MERI or the OOPS, were additional prognostic factors in our study.

A recent study compared the OOPS and MERI indices in 68 patients who underwent ossiculoplasty [15]. They showed that MERI was a better indicator of a successful surgery, defined as ABG ≤ 20 dB, than OOPS [15]. More importance is given to ossicle status in MERI than in OOPS. They suggested that the superiority of MERI is associated with the importance of ossicle status; however, our results found that the opposite was true. This may be because of decreased importance of ossicle status. The study of Kotzias et al. enrolled patients between 2006 and 2016. Although their data did not present the material used for ossiculoplasty, an artificial prosthesis made of materials; such as hydroxyapatite or titanium with a more biocompatible and proper design, may not have been fully developed during the period of whole their study. Ossicle status is an important prognostic factor when the patient does not have a proper prosthesis, which may lead to the superiority of the MERI over the OOPS. Additionally, the study included a small number of patients, and most patients were at intermediate risk. There were only three patients at high risk according to the OOPS. In our study, all patients had a autologus ossicle or a proper prosthesis and it led to a decrease in the importance of ossicle status compared to results using old cohort. The importance of mastoidectomy as an additional prognostic factor and decreased importance of ossicle status were both reflected in the OOPS.

One of the major limitations of the scoring systems was that they calculated the same score for different conditions. Furthermore, these two scoring systems measure scores for each variable using simple dichromatic or trichromatic approaches. For example, dry perforation and the presence of cholesteatoma are scored as 1. However, the importance of these two conditions would be very different according to the specific type of COM or severity/nature of each variable. Moreover, the scoring systems did not consider variables that were important factors in determining the types of COM; they apply equally to each type of COM. Important risk factors for suppurative, cholesteatomatous, or adhesive otitis media are the duration of otorrhea and pathogen species, position and extension of cholesteatoma, and patency of the eustachian tube and severity of adhesion, respectively. However, these were not considered in the two scoring systems. Some centers may not routinely use these scoring systems because of these limitations. Our center also did not use the OOPS or MERI scoring systems, but various factors associated with prognosis were collected from medical charts. Before the operation, clinical factors, such as otorrhea, smoking status, or underlying comorbidities were described, and photographs of the tympanic membrane were captured using endoscopy. In addition, the status of the ossicle and the presence or absence of cholesteatoma were routinely described in the operation records. These are well-known traditional factors associated with postoperative hearing or prognosis in patients with COM. An individualized approach, according to the presence or absence of each indicator was performed at our center. We compared two scoring systems, and our results would be useful as a bridge study before developing more accurate scoring systems according to the specific type of COM.

## Limitations

There were a few limitations to this study. First, this study had a retrospective design with a single center and ethnicity. The two scores were calculated using medical records that were independently collected; therefore, the score calculated using them may not completely reflect the status of each variable. Additionally, the type of surgery was selected based on clinical indication regardless of the scoring system. Additionally, in the comparison of some variables such as presence of diabetes mellitus and ossiculoplasty materials, the difference between sample size limited the analysis. Second, our study included a relatively large sample size with long-term follow-up. However, 12 months is insufficient to investigate the extrusion of artificial material. Therefore, a longer follow-up period with multi-center studies is warranted. Third, our study did not use high-frequency audiograms as outcome measurements. The mean age of the patients was 53.2 ± 15.4 years old. Relatively old patients may be prone to a increased hearing threshold at high frequencies, regardless of ear disease. Therefore, the change in hearing at high frequencies after the operation may be less clear than that at low frequencies. Furthermore, both the 1995 American Academy of Otolaryngology–Head and Neck Surgery, Committee on Hearing and Equilibrium consensus guidelines and the Korean Society of Otology guidelines recommend that the success of the surgery can be evaluated using air gain, change of high-tone bone conduction, air-bone gap closure, or postoperative air conduction without a high-frequency audiogram. Fourth, in our study, the graft was evaluated using endoscopy alone. A tympanogram is more useful for diagnosing complications such as re-perforation or adhesions, but this is not routinely evaluated in our center. In addition, the patency of the Eustachian tube was assessed using medical records and intraoperative microscopic findings. However, this is not an accurate method for evaluating the patency of the Eustachian tube, such as dynamic motion. At our center, physical examinations using endoscopy and pure-tone audiometry are routinely evaluated during the postoperative period. If middle ear conditions, such as effusion or inflammation, ossicle chain discontinuity, and tympanic membrane perforation are suspected upon physical examination or pure-tone audiometry, we performed tympanogram and temporal bone CT. This step-by-step approach alone could be sufficient to assess complications without routinely specific evaluations.

## Conclusion

In conclusion, compared with MERI, the OOPS index was more closely associated with the hearing outcomes, which may be due to the extent of inflammation in the OOPS index. The modified index, including smoking and comorbidities, among variables in OOPS, is considered to be a good scoring system for predicting hearing outcomes in patients who underwent tympanoplasty.

## Supporting information

S1 Fig The proportions of three groups in two indices. The number of patients in the mild, moderate, and severe MERI groups was 283, 158, and 85, respectively. Regarding the mild group for MERI, the number of patients in the low, intermediate, and high OOPS groups was 163 (57.6%), 85 (30.0%), and 35 (12.4%), respectively. Regarding the moderate group for MERI, the number of patients in the low, intermediate, and high OOPS groups was 68 (43.0%), 65 (41.1%), and 25 (15.8%), respectively. Regarding the high group for MERI, the number of patients in the low, intermediate, and high OOPS groups was 32 (37.6%), 26 (30.6%), and 27 (31.8%), respectively. MERI, middle ear risk index; OOPS, ossiculoplasty outcome parameter staging.
(TIF)

S1 Table MERI and OOPS scoring. (DOCX)

S2 Table Comparison of hearing outcomes according to groups of two indices. (DOCX)

S3 Table Comparisons of success rate according variables without two indices. (DOCX)
