# Supplementary figures and images for: Prediction of hearing outcomes in chronic otitis media patients underwent tympanoplasty using ossiculoplasty outcome parameter staging or middle ear risk indices
Source: PLoS One. 2021 Jul 29;16(7):e0252812. doi: 10.1371/journal.pone.0252812 (PMC8321221; doi:10.1371/journal.pone.0252812)

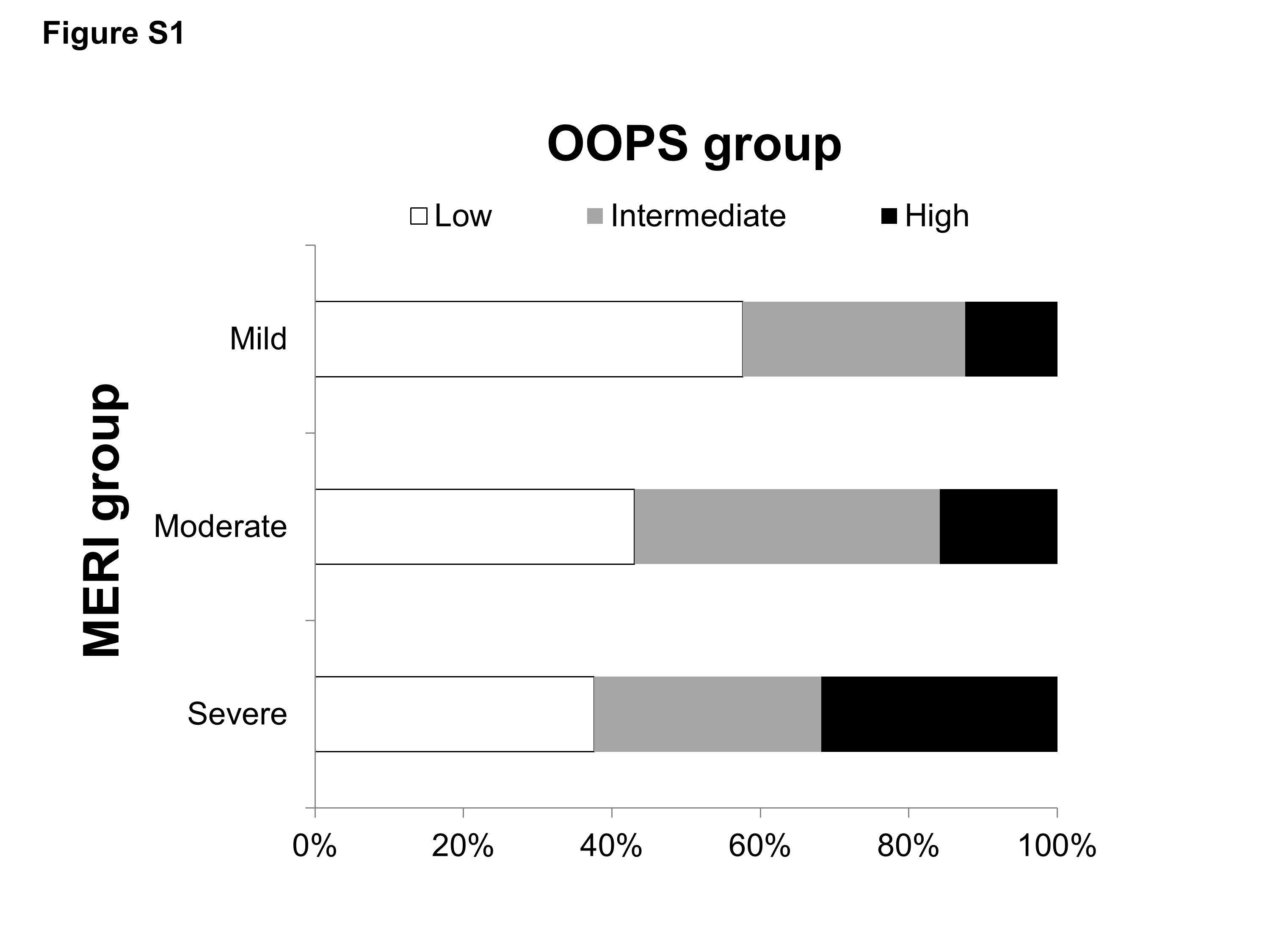

Supplement: S1 Fig — The number of patients in the mild, moderate, and severe MERI groups was 283, 158, and 85, respectively. Regarding the mild group for MERI, the number of patients in the low, intermediate, and high OOPS groups was 163 (57.6%), 85 (30.0%), and 35 (12.4%), respectively. Regarding the moderate group for MERI, the number of patients in the low, intermediate, and high OOPS groups was 68 (43.0%), 65 (41.1%), and 25 (15.8%), respectively. Regarding the high group for MERI, the number of patients in the low, intermediate, and high OOPS groups was 32 (37.6%), 26 (30.6%), and 27 (31.8%), respectively. MERI, middle ear risk index; OOPS, ossiculoplasty outcome parameter staging. (TIF) [file pone.0252812.s001.tif]
