# Supplementary material for: Prediction of hearing outcomes in chronic otitis media patients underwent tympanoplasty using ossiculoplasty outcome parameter staging or middle ear risk indices
Source: PLoS One. 2021 Jul 29;16(7):e0252812. doi: 10.1371/journal.pone.0252812 (PMC8321221; doi:10.1371/journal.pone.0252812)
Supplement: S1 Table — (DOCX) [file pone.0252812.s002.docx]

|  | MERI | | OOPS | |
| --- | --- | --- | --- | --- |
|  | Status | Score | Status | Score |
| Otorrhea | Dry | 0 | None | 0 |
|  | Sometimes humide | 1 | Present > 50% of the time | 1 |
|  | Always humid | 2 |  |  |
|  | Humid, cleft palate | 3 |  |  |
| Perforation | Absent | 0 | – |  |
|  | Present | 1 |  |  |
| Cholesteatoma | Absent | 0 | – |  |
|  | Present | 1 |  |  |
| Ossicular status | M +, I +, S + | 0 | Normal | 0 |
|  | M +, S + | 1 | M + | 1 |
|  | M +, S – | 2 | M – | 2 |
|  | M –, S + | 3 |  |  |
|  | M –, S – | 4 |  |  |
|  | Ossicle head fixation | 2 |  |  |
|  | Stapes fixation | 3 |  |  |
| Middle ear (granulation or effusion) | No | 0 | Normal | 0 |
|  | Yes | 1 | Fibrotic | 2 |
| Previous surgery | None | 0 | No | 0 |
|  | Staged | 1 | Revision | 2 |
|  | Revision | 2 |  |  |
| Smoker | No | 0 | – |  |
|  | Yes | 2 |  |  |
| Mastoidectomy | – |  | No mastoidectomy | 0 |
|  |  |  | CWU mastoidectomy | 1 |
|  |  |  | CWD mastoidectomy | 2 |

Abbreviations: MERI, middle ear risk index; OOPS, ossiculoplasty outcome parameter staging; M +, malleus present; M –, malleus absent; I +, incus present; S +, stapes present; S –, stapes absent; CWU, canal wall up; CWD, canal wall down
