# Supplementary material for: Prediction of hearing outcomes in chronic otitis media patients underwent tympanoplasty using ossiculoplasty outcome parameter staging or middle ear risk indices
Source: PLoS One. 2021 Jul 29;16(7):e0252812. doi: 10.1371/journal.pone.0252812 (PMC8321221; doi:10.1371/journal.pone.0252812)
Supplement: S2 Table — (DOCX) [file pone.0252812.s003.docx]

|  | MERI | | | | OOPS | | | |
| --- | --- | --- | --- | --- | --- | --- | --- | --- |
|  | Mild | Moderate | Severe | *P*-value | Low | Intermediate | High | *P*-value |
| 3 M |  |  |  |  |  |  |  |  |
| PostABG | 16.8 ± 14.8 | 17.8 ± 13.9 | 16.8 ± 12.2 | 0.759 | 12.5 ± 11.4 | 21.0 ± 14.5* | 23.2 ± 15.9* | <0.001 |
| ABG closure | 3.4 ± 11.0 | 4.2 ± 10.2 | 3.8 ± 10.2 | 0.752 | 3.9 ± 9.3 | 3.6 ± 11.8 | 3.2 ± 12.3 | 0.865 |
| △Ht-BC | 0.13 ± 8.87 | 0.11 ± 8.84 | 1.24 ± 7.98 | 0.559 | -0.54 ± 8.25 | 1.14 ± 8.44 | 1.17 ± 10.3 | 0.084 |
| AG | 12.2 ± 16.1 | 12.2 ± 15.6 | 12.4 ± 13.4 | 0.991 | 9.7 ± 12.5 | 13.7 ± 17.1 | 16.8 ± 18.7* | <0.001 |
| PostopAC | 43.3 ± 23.9 | 46.0 ± 23.2 | 44.7 ± 22.2 | 0.494 | 35``.1 ± 18.1 | 52.1 ± 24.2* | 56.7 ± 25.0* | <0.001 |
| PostopSDT | 91.6 ± 23.7 | 91.1 ± 22.5 | 95.5 ± 13.1 | 0.299 | 96.8 ± 13.0 | 88.4 ± 25.8* | 85.2 ± 30.7* | <0.001 |
| 12 M |  |  |  |  |  |  |  |  |
| PostABG | 16.0 ± 16.4 | 17.7 ± 14.8 | 16.1 ± 13.3 | 0.500 | 12.1 ± 13.3 | 20.2 ± 16.4* | 22.6 ± 15.8* | <0.001 |
| ABG closure | 3.0 ± 11.5 | 4.1 ± 11.8 | 3.0 ± 11.5 | 0.602 | 3.4 ± 10.4 | 3.9 ± 12.6 | 1.9 ± 12.7 | 0.412 |
| △Ht-BC | 1.34 ± 11.11 | 0.40 ± 9.49 | 2.61 ± 8.72 | 0.276 | 0.60 ± 9.69 | 1.48 ± 10.66 | 2.82 ± 11.19 | 0.208 |
| AG | 12.0 ± 17.6 | 13.0 ± 16.6 | 13.3 ± 13.9 | 0.761 | 10.3 ± 14.2 | 13.6 ± 19.0 | 16.9 ± 18.1* | 0.003 |
| PostopAC | 43.0 ± 24.0 | 45.9 ± 23.2 | 44.2 ± 22.4 | 0.461 | 35.1 ± 18.9 | 51.6 ± 23.9* | 56.3 ± 24.5* | <0.001 |
| PostopSDT | 91.8 ± 23.4 | 91.4 ± 22.4 | 94.1 ± 17.6 | 0.628 | 96.6 ± 13.2 | 88.2 ± 25.8* | 85.2 ± 31.4* | <0.001 |

The data are expressed as the means ± standard deviations. *P*-values are tested using one-way analysis of variance, followed by a post hoc Tukey’s comparison for continuous variables. **P* < 0.05 vs. mild or low risk group.

Postop, postoperative; ABG, air bone gap; △Ht-ABG, change of high-tone bone conduction; AC, air conduction; SDT, speech discrimination test; OOPS, ossiculoplasty outcome parameter staging; MERI, middle ear risk index; 3M, 3 months after operation; 12M, 12 months after operation.
