# Supplementary material for: Prediction of hearing outcomes in chronic otitis media patients underwent tympanoplasty using ossiculoplasty outcome parameter staging or middle ear risk indices
Source: PLoS One. 2021 Jul 29;16(7):e0252812. doi: 10.1371/journal.pone.0252812 (PMC8321221; doi:10.1371/journal.pone.0252812)
Supplement: S3 Table — (DOCX) [file pone.0252812.s004.docx]

**Table S3. Comparisons of success rate according variables without two indices**

|  | Total numbers | Success (3M) | *P*-value | Success (12M) | *P*-value |
| --- | --- | --- | --- | --- | --- |
| Age |  |  | 0.121 |  | 0.155 |
| <55 years old | 259 | 226 (87.3%) |  | 225 (86.9%) |  |
| ≥55 years old | 267 | 220 (82.4%) |  | 220 (82.4%) |  |
| Eustachian tube |  |  | 0.305 |  | 0.509 |
| Patent | 417 | 357 (85.6%) |  | 355 (85.1%) |  |
| Obstructed | 109 | 89 (81.7%) |  | 90 (82.6%) |  |
| Diabetes mellitus |  |  | 0.566 |  | 0.332 |
| Absence | 482 | 410 (85.1%) |  | 410 (85.1%) |  |
| Presence | 44 | 36 (81.8%) |  | 35 (79.5%) |  |
| Hypertension |  |  | 0.070 |  | 0.014 |
| Absence | 421 | 351 (83.4%) |  | 348 (82.7%) |  |
| Presence | 105 | 95 (90.5%) |  | 97 (92.4%) |  |
| Ossiculoplasty |  |  | <0.001 |  | <0.001 |
| No | 221 | 208 (94.1%) |  | 204 (92.3%) |  |
| Columellization | 275 | 214 (77.8%) |  | 219 (79.6%) |  |
| Interposition | 30 | 24 (80.0%) |  | 22 (73.3%) |  |
| Ossiculoplasty materials |  |  | 0.020 |  | 0.045 |
| Incus | 28 | 17 (60.7%) |  | 18 (64.3%) |  |
| Hydroxyapatitie or titanium | 277 | 221 (79.8%) |  | 223 (80.5%) |  |

Data are expressed as numbers (percentages). Statistical analyses are performed using the chi-square test.

Abbreviations: 3M, 3 months after surgery; 12M, 12 months after operation.
